# Wear Scar Similarities between Retrieved and Simulator-Tested Polyethylene TKR Components: An Artificial Neural Network Approach

**DOI:** 10.1155/2016/2071945

**Published:** 2016-08-14

**Authors:** Diego A. Orozco Villaseñor, Markus A. Wimmer

**Affiliations:** ^1^Orthopedic Surgery, Rush University Medical Center, 1611 W. Harrison Street, Chicago, IL 60612, USA; ^2^Bioengineering, University of Illinois at Chicago, 851 S. Morgan Street, Chicago, IL 60607, USA; ^3^Departamento de Bioingeniería, Tecnológico de Monterrey, Campus Guadalajara, Guadalajara, JAL, Mexico

## Abstract

The aim of this study was to determine how representative wear scars of simulator-tested polyethylene (PE) inserts compare with retrieved PE inserts from total knee replacement (TKR). By means of a nonparametric self-organizing feature map (SOFM), wear scar images of 21 postmortem- and 54 revision-retrieved components were compared with six simulator-tested components that were tested either in displacement or in load control according to ISO protocols. The SOFM network was then trained with the wear scar images of postmortem-retrieved components since those are considered well-functioning at the time of retrieval. Based on this training process, eleven clusters were established, suggesting considerable variability among wear scars despite an uncomplicated loading history inside their hosts. The remaining components (revision-retrieved and simulator-tested) were then assigned to these established clusters. Six out of five simulator components were clustered together, suggesting that the network was able to identify similarities in loading history. However, the simulator-tested components ended up in a cluster at the fringe of the map containing only 10.8% of retrieved components. This may suggest that current ISO testing protocols were not fully representative of this TKR population, and protocols that better resemble patients' gait after TKR containing activities other than walking may be warranted.

## 1. Introduction

Wear performance evaluation has become an important preclinical tool for the assessment of materials and designs of total knee replacement (TKR) components. To date, the International Organization for Standardization (ISO) has established two wear testing protocols to evaluate the long-term wear performance of TKR components [1, 2]. Both ISO protocols aim at replicating load and motion characteristics of a natural knee during level walking, which is considered to be the most frequently performed physical activity of daily living [3]. As with any simulation tool, the ultimate goal of wear simulations is to recreate in vivo conditions as closely as possible. For knee wear simulation, this means recreating wear damage characteristics (wear rates, wear modes, wear patterns, damage appearances, particle sizes, and morphologies) that are similar to those generated in vivo. However, reproducing in vivo wear damage characteristics of the knee has proven to be very challenging because simulators generate tibial liner wear scars that are less variable in size and location compared to those observed in retrievals of the same design type [4, 5].

Several factors, such as the characteristics of the prosthesis (materials and designs), the patient (height, weight, joint loading during daily activities, and activity level), and the surgical technique (alignment and soft tissue balancing), influence the wear of a TKR polyethylene tibial liner. Discrepancies between simulated and in vivo worn components can be identified by comparing their wear scar characteristics, which are substantially influenced by the kinetics and kinematics of the knee joint. Hence, wear scars are useful indicators of the physiological load and motion spectrum applied to the tibial insert during daily physical activity. However, a detailed analysis of wear scars is very complex. The mathematical description of wear scar patterns is nonlinear and multidimensional, which makes it very difficult or even impossible to model these patterns using traditional mathematical or statistical methods. For instance, different geometric parameters, including area, perimeter, or centroid of a wear scar, could be used to form the basis for a specific model. However, even multiple geometric parameters may not sufficiently explain the overall wear scar generation process, which is why we propose to analyze in vivo and in vitro generated war scars as a whole using bitmap images.

In this study, an artificial neural network (ANN) model based on image information is implemented as a data mining tool to differentiate wear scars that originate from different loading histories. ANNs have been successfully used for similar models because of their ability to handle nonlinear behavior, to learn from experimental data, and to generalize solutions [6–11]. From the pool of ANN models, the self-organizing feature map (SOFM) was selected for this study because it is an unsupervised neural network (i.e., no a priori knowledge of the data structure and classification is used). It is frequently used for the visualization of high dimensional data and for data mining and knowledge discovery [7–10, 12–14]. SOFMs are particularly useful because of their ability to map nonlinear statistical relationships between high dimensional data onto a convenient and easily comprehendible two-dimensional map. This type of mapping preserves the topology of the data, meaning that points within close proximity in the high dimensional space are mapped to neighboring map units in the output space. While this modeling technology has been used for image mapping since the early 2000s [15], to the best of our knowledge, it has not been used for applications in orthopedic tribology.

The purpose of the present investigation was to create a clustering structure of wear scar images based on similarities between retrieved (revision and postmortem) and simulator-tested components of the same design type. Wear scars from the retrieved group were used to create a clustering structure, whereas the wear scars from simulator-tested components were then assigned to the existing clustering structure based on their similarities. Subsequently, data mining was performed to understand the similarities among wear scars clustered together as well as to explain the differences between wear scars of different clusters. Two hypotheses were tested: (1) wear scars from retrieved components will generate several clusters of wear scars because of the variability of wear scar size and location that characterizes retrieved components and (2) all simulator components, regardless of the testing standard used, will be clustered together, reflecting the comparability of the two ISO testing standards and their limitation in generalizing the greater variability observed in retrieved components of the same design type.

## 2. Materials and Methods

**Figure 1:**
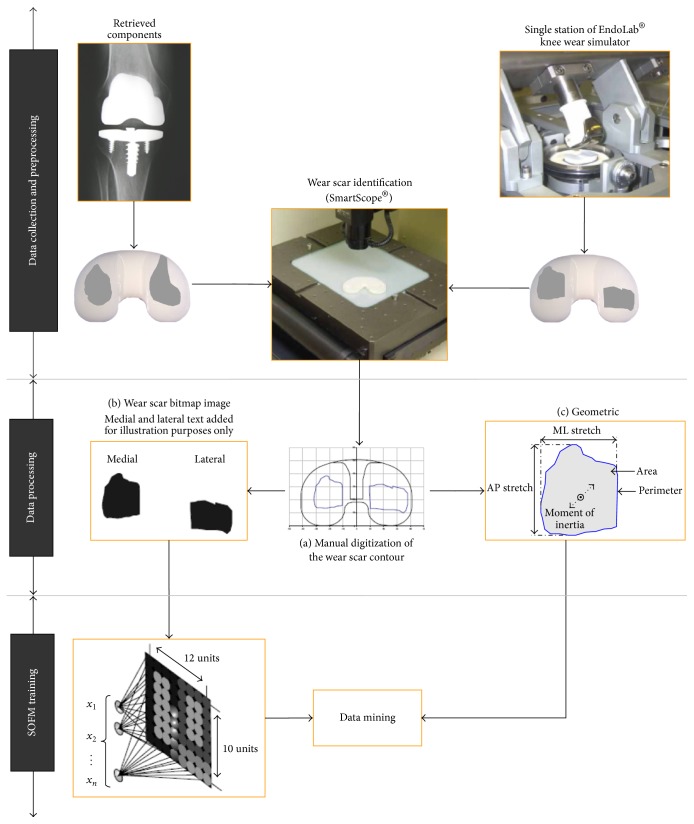
Flow diagram providing the methodology applied in this investigation. The methodology was divided into three main sections: (1) data collection and preprocessing; (2) data processing; and (3) SOFM training.

An overview of the materials and methods used in this investigation is presented in Figure 1.

With approval from the Institutional Review Board (#L03072801), twenty-one postmortem- and fifty-four revision-retrieved tibial liners were selected from the Retrieval Repository at Rush University Medical Center (Table 1). Before being included in the study, components were screened for missing demographic information and for signs of heavy delamination. All retrieved components were manufactured by a single company (Zimmer, Inc., Warsaw, IN, USA) and were of the posterior cruciate retaining MG-II design, a fixed bearing prosthesis with a flat tibial polyethylene plateau.

Wear testing was performed using eight tibial liners, which were of the same design type and company as the retrieved components (MG-II, Zimmer, Inc., Warsaw, IN, USA). Testing components were randomized into two equal groups. In each group, three samples were tested for wear performance and one sample served as a loaded soak control. The tibial plateaus were machined from ultra-high molecular weight polyethylene (UHMWPE), gamma sterilized, and packaged in a nitrogen environment by the manufacturer. The boxes were opened immediately prior to testing.

Wear performance tests were carried out in a four-station knee simulator (EndoLab, Rosenheim, Germany). The simulator met ISO standards [1, 2] and could be set up to run either in load control mode or in displacement control mode. The simulator motions were hydraulically actuated and closed-loop controlled. The difference in control mode refers to two degrees of freedom (anterior-posterior and internal-external, resp.) that were either load or displacement controlled, resulting in different implant articulations that were determined by the specific design aspects of the artificial joint.

The wear tests were conducted prior to 2009 following the original ISO standards and have been published elsewhere [16]. Briefly, each simulator station was comprised of a temperature-controlled chamber that maintained the test lubricant at 37°C. The lubricant was based on a buffered mixture of bovine serum (Hyclone Inc., Logan, UT, USA) mixed with a physiological salt solution to achieve a final protein content of 30 g/L and a pH of 7.4. In order to sequester metal ions, 200 mg/L ethylenediaminetetraacetic acid (EDTA) was added. All chambers were closed and sealed during the entire test to minimize fluid evaporation and contamination. The simulator was connected to a computer equipped with a user interface for machine control, test supervision, and data acquisition.

The first simulator group was tested in load control mode (LCM) and the second group was tested in displacement control mode (DCM). The LCM and DCM tests followed the same general protocol and testing parameters stated in the original 2002 and 2004 versions [1, 2]. Tests were conducted at 1.0 Hz cycle frequency and lasted for five million cycles (Mc). Load and displacement input represented one full walking cycle per test cycle and were taken from the respective ISO standards. The experiment was interrupted every 0.5 Mc to dismount, clean, and weigh the specimens according to the ISO standard [17]. Wear scars on the tibial UHMWPE plateaus that developed during the test were analyzed after test completion.

Medial and lateral articulating surfaces were visually analyzed using a video-based microscope (SmartScope, OGP NY, USA). Wear scars were digitized by manually tracking their contours (i.e., the boundary between worn and unworn areas) on the liner surface (Figure 1(a)) [18]. Since the goal of this study was to compare wear scar patterns using images rather than discrete geometric parameters, black and white wear scar bitmap images (220 × 170 pixels) were generated for each component (Figure 1(b)). Each bitmap image contained medial and lateral wear scar shapes with black pixels representing worn areas and white pixels representing unworn areas. Each bitmap image was converted to a 220 × 170 matrix with “1” representing white pixels and “0” representing black pixels. Each matrix was then reshaped to a single row vector and used as input for the SOFM model. While the component border was not kept in the image, the length and height of the image were adjusted to match the component size. All components were normalized to an equal size and right implantation side. Components with unknown implantation side (~7%, Table 1) were normalized after side determination with ANN. Geometric wear scar parameters, including area, perimeter, centroid, bounding box, anterior/posterior stretch, medial/lateral stretch, moment of inertia, and multiple shape factors, were computed for each component (Figure 1(c)) and used for statistical analysis.

**Figure 2:**
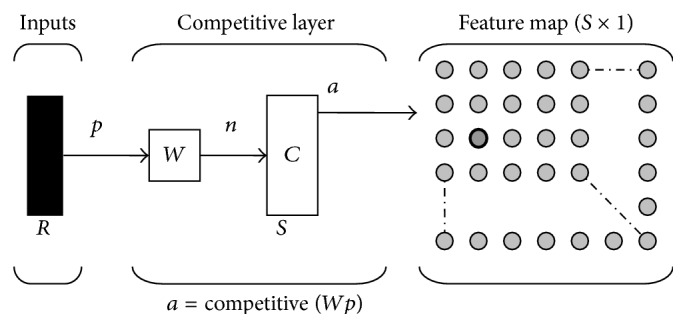
Self-organizing feature map (SOFM) neural network structure. In the competitive layer, input vectors are assigned to the neuron with the shortest Euclidean distance. Similar input vectors will be assigned to neighboring neurons.

The SOFM network was designed and trained using the Matlab SOM Toolbox 2.0 (Helsinki University of Technology, Finland). A sensitivity analysis was conducted to identify ideal training parameters generating best mapping results. The networks consisted of an input layer of 37,400 neurons (from image dimensions of 220 × 170 pixels = 37,400), a competitive layer, and an *n* × *m* neurons map or output layer (Figure 2). Five different networks with different map dimensions were generated. Map size and neighborhood radius were the only parameters tuned during the sensitivity analysis. The learning rate was linearly adjusted for all networks and the presentation of training samples was done in a random order. Training was performed using postmortem-retrieved components only. Subsequently, simulator- and revision-retrieved components were assigned to already existing clusters. No network learning occurred from the simulator wear scar patters. Training was done using the batch algorithm based on the Euclidean metric. Statistical analysis of the clustering structure was performed only from the map providing the smallest quantization error (which is a measure of “fit” between input and output mapping) and a well-defined cluster structure.

The u-matrix method was used to visualize the distance of each map neuron to its neighbors. The shorter the distance between neurons, the smaller the difference between them [19, 20]. This method was used to visually uncover the clustering structure in the SOFM. Commonly, a two-dimensional color coded u-matrix is used to identify cluster boundaries. Component planes (another commonly used visualization tool) were not created because the type of input data used in this study would have produced 37,400 component planes (one for each dimension).

Clustering robustness was evaluated by producing multiple versions of the map with the best mapping results. The goal of this process was to detect mapping irregularities caused by the inherent mapping error meaning that data from a high dimensional space mask a significantly smaller dimensional space. To detect clustering irregularities, three network versions were created and trained until they converged. The networks were created and analyzed by an independent investigator. The networks' map size, learning rate, and neighborhood radius were left unchanged. The only training parameters that differed between networks were the initial values of the map neurons and the presentation of the training samples, which were both randomly chosen. The clustering structure was visualized and compared between network versions. The map neurons assigned to each wear scar in each of the networks were recorded and used for comparison. Cohen's kappa analysis was carried out to investigate if each component was consistently clustered with the same group of components.

SOFM mapping configurations were evaluated based on quantization errors. To test the interrater reliability of the network, intraclass correlation coefficients (ICC) were computed. An analysis of variance (ANOVA) was conducted to detect differences within and among clustered wear scar images. The geometric parameters, computed for medial and lateral wear scars separately, were used as output variables in the statistical analysis. The associations between two available input variables (“time in host” and “age at surgery”) with output variables were evaluated using regression analysis. Only clusters with available input on more than three retrieved components were included. The chance probability that five of six simulator components would land in a single cluster was estimated using the binomial distribution. The probability of “success” (i.e., landing in cluster “X”) was estimated from the proportion of revision and postmortem components that landed in that cluster. All statistical analyses were performed in SPSS 16.0 for Windows (SPSS Inc., Champaign, IL, USA).

## 3. Results

A network with a map size of 12 × 10 and initial to final neighborhood radii of 4 to 1 was found to provide the lowest quantization error (*q*_*e*_ = 11.14) and a well-defined clustering structure. The other network configurations evaluated were 20 × 10/4 to 1, 20 × 10/4 to 1, 10 × 10/4 to 1, 10 × 10/5 to 3.5, and 7 × 7/4 to 1. The 20 × 10 network had a lower quantization error (*q*_*e*(20×10)_ = 10.9) than the network selected for the final analysis; however, its cluster boundaries were not easily identifiable. The remaining networks evaluated had higher quantization errors: *q*_*e*(10×10/4 to 1)_ = 12.7; *q*_*e*(10×10/5 to 3.5)_ = 15.3; and *q*_*e*(7×7)_ = 17.1.

The clustering robustness analysis showed substantial interrater reliability for the different SOFMs created with a kappa value of 0.69 (*p* < 0.001) and 95% CI (0.667, 0.712). Despite the random initial values of map neurons and the random presentation of the training samples, tibial inserts that were clustered together in the first round stayed mostly in the same cluster during the second round. On average 84% (SD ± 19%) of all components were consistently mapped with the same components.

**Figure 3:**
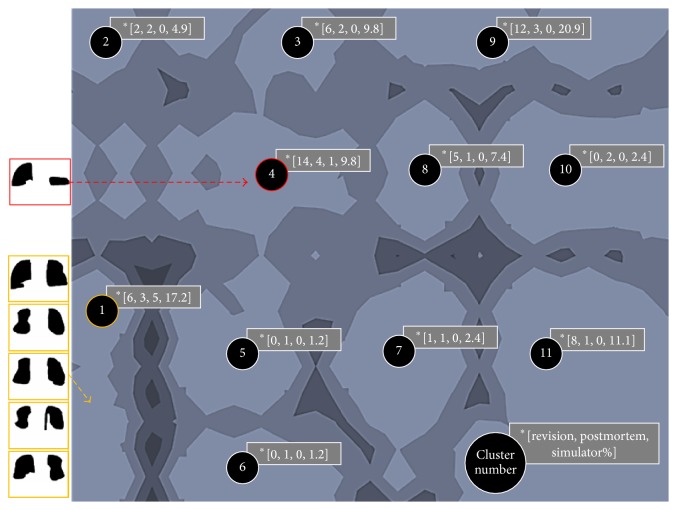
U-matrix visualization of the SOFM after training. Eleven wear pattern clusters were identified. Five out of six in vitro tested components were assigned to cluster “1”. ^*∗*^The number of revised (R), postmortem (P), and simulator (S) components and the total percentage (%) of components assigned to each group are noted in brackets [R, P, S, %]. Light map colors represent cluster areas (valleys), while darker colors represent cluster boundaries (hills).

**Figure 4:**
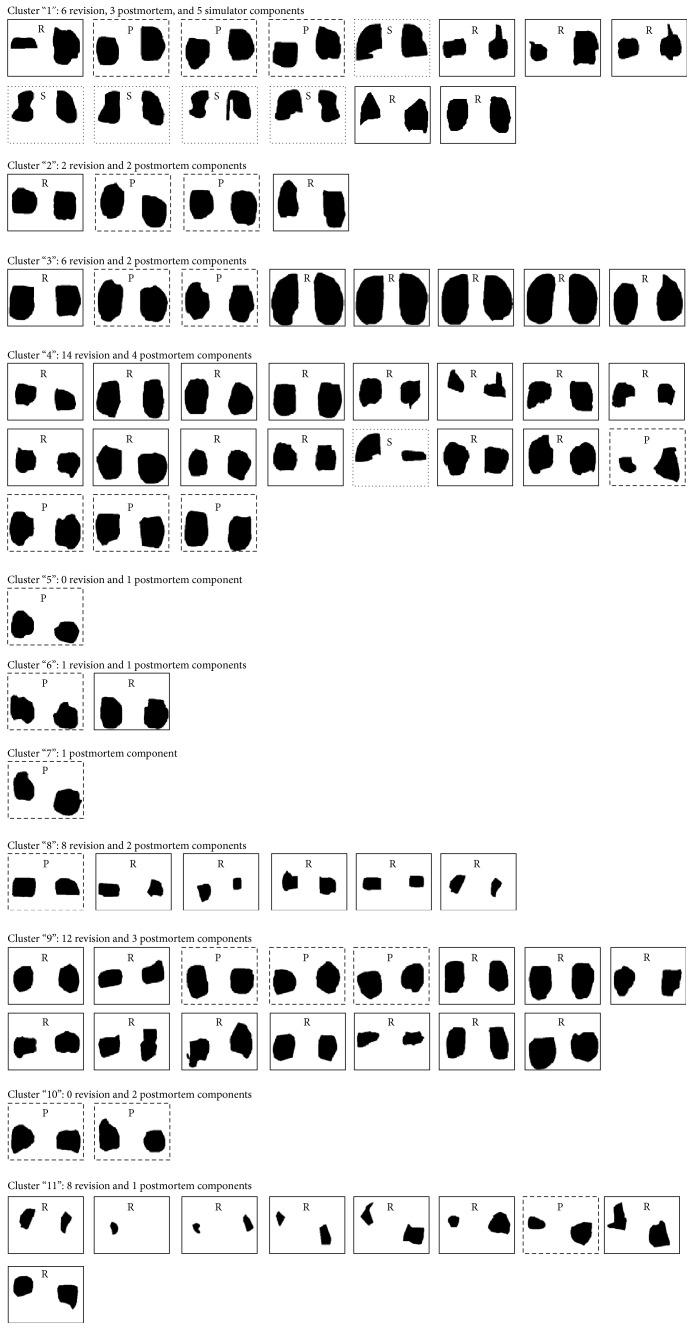
Eleven Clusters were established. Except for one, all simulator components fell in Cluster “1” together with six revision and three postmortem components.

Using the u-matrix visualization method, eleven clusters became evident, each containing at least one postmortem-retrieved component and a maximum of 18 retrieved components (Figures 3 and 4). While 54 revision-retrieved components were assigned to nine of eleven clusters, all but one of the six simulator-tested components were placed in cluster 1. The chance probability that five or more of the simulator components would land in cluster 1 was estimated to be 1.6*E* − 4 using the binomial distribution. It is worth mentioning that cluster 1 contained only 10.8% of retrieved components and was one of the more isolated clusters at the fringe of the map.

The geometric features of the wear scars are summarized in Table 2. There was no single geometric variable that could have explained the differences between all clusters. Thus, it was found that cluster 1 was not significantly different from the other clusters based on wear scar geometric parameters alone, although the SOFM network had established cluster 1 as one of the most dissimilar clusters. Interestingly, the largest number of significant differences was found in cluster 11. For simulator components only, medial and lateral wear scars were more anteriorly located and more symmetrical than for the retrieved components in the cluster. However, only the anterior location differed significantly from all other cluster-retrieved components (*p* < 0.05), whereas the wear scar symmetry did not. The associations between two available input variables (i.e., “time in host” and “age at surgery”) and geometric output variables differed between the various groups (Table 2).

## 4. Discussion

In this study, the relationship between wear scar images of simulator-tested and retrieved TKR tibial components was investigated. A nontraditional qualitative modeling approach was used to project nonlinear relationships of a high dimensional data set (wear scar images) onto a two-dimensional map. The SOFM algorithm was used as a data mining and knowledge discovering tool and served as visual aid in the discovery of wear scar characteristics.

After successfully training with wear scars from postmortem-retrieved components, eleven clusters were created. Purposefully, postmortem-retrieved inserts were used for this training purpose since they count as well-functioning at the time of retrieval and as such may be considered a “gold standard” for TKR wear simulation. As hypothesized, several clusters of wear scars were generated, mimicking the variability of wear scar patterns that characterizes retrieved components [4, 5]. Further, wear scars generated through mechanical simulation were clustered together, suggesting that the clustering process is meaningful in that wear scars of a similar loading history are recognized by the SOFM. It must be stressed that this cluster contained wear scars from both load and displacement control tested inserts, which showed distinct differences in wear scar size in an earlier study [17]. Hence, there must be other important wear scar features that render them similar.

All but one of the simulator-tested components were clustered together. The simulator-tested component assigned to cluster 4 clearly differs visually from the other simulator components (see Figure 3). We were aware of this difference because one of the AP actuators of the simulator became faulty during one of the wear tests. However, this information was not used as input into the SOFM. The only data and information used as input into the network was the medial and lateral wear scar images from both retrieved and simulator-tested components, which were all presented to the network in a random order during the training process. Hence, it appears that the SOFM network is capable of identifying subtle differences in loading history.

Based on the clustering results, the load and displacement control tested inserts account only for about 11% of the wear scar characteristics found in retrieved components. Cluster 1 is at the fringe of the cluster map and relatively isolated from other components (as indicated by the high ridge around it; see Figure 3). Ideally, the cluster containing the simulator components establishes itself in the center of the map to have shorter distances to all components and, thus, be more representative. The sole application of ISO gait cycles may not be sufficient in mimicking the greater variability of wear scar patterns observed on retrieved components. Ngai et al. reported that not only do the motion patterns of TKR patient differ from the motion pattern applied by the displacement [21] and/or load control [22] standard, but they are also highly variable between patients [23]. Also, these findings may indicate that it is important to consider other activities of daily living for knee wear testing. Both Benson et al. [24] and Cottrell et al. [25] found that the inclusion of one cycle of stair descent or ascent for every seventy cycles of level walking during wear testing produced more in vivo-like wear scars than those generated by walking alone. Thus, the variability of wear scars observed in retrieved components may not just be the result of different walking patterns but may reflect the range of physical activities performed by the patient, raising the need for a more representative TKR motion testing pattern.

There are limitations to using the SOFM. The network does not identify variables that characterize each cluster and best discriminate between the clusters [7]. Hence, the user is left in ambiguity. In this study we were unable to explain wear scar clustering by geometric characteristics. Since the clustering created by the SOFM is a projection of a nonlinear and high dimensional input space, the clustering results may not be fully explained by traditional linear statistical models. Perhaps, future mathematical means may resolve this issue. Because of the nature of the clustered data of this study, the issue was amplified. Typically, cluster correlations created by a SOFM are performed using component planes; however, our data sets were based on pixel information and this analysis was not applicable. A second limitation was that the high dimensionality of the input data set affected the training time of the SOFM, ranging from four hours to almost a full day until convergence, depending on the map size. Smaller bitmap images or a different representation of the wear scar pattern may be used to limit the computational time spent on training the SOFM. Smaller bitmap images may also reduce the quantization error because this error depends directly on the dimensionality of the input space and the output map where a greater dimensionality reduction will result in a greater quantization error. On the other hand, a coarser, more pixilated wear scar may result in loss of sensitivity and a threshold has yet to be established. Finally, there were also limitations with the study design. The simulator tests were executed according to the original knee wear testing standards and should be repeated following the updated protocols. Our retrieval collection was small in size, with modest and partially incomplete patient information. This resulted in underrepresented clusters with few components and prevented a thorough data mining. Both “time in situ” and “patient age” are only auxiliary variables for prosthetic use and patient activity. Knowledge about the number of individual walking steps, the specific gait mechanics, and activity profile of each patient may have provided important clues in identifying associations and differences within and between clusters.

## 5. Conclusions

In conclusion, an artificial neural network approach has been applied for the comparison of wear scar images of simulator and retrieved TKR tibial inserts. This modeling approach proved to be robust and repeatable. The model, which was based on the self-organizing feature map network, can be used to directly compare wear scars from simulator and retrieved tibial liners. The SOFM network analysis revealed that (1) wear scars from retrieved components are highly variable, generating multiple clusters, (2) wear scars generated through wear testing using two different ISO standards were clustered together and are, thus, deemed comparable, and (3) wear scars from simulator components were clustered away from the center of the map and, therefore, are not representative of the whole retrieval collection. In the future, we may check if a new multiactivity testing protocol is capable of generating wear scars that more closely resemble retrieved components. The SOFM model may also be used for data mining of very large retrieval cohorts and search for associations and differences beyond physical context. For example, the input could contain surgical factors and/or socioeconomic factors. In the summary, the SOFM established in this study provides a unique and versatile platform for future discovery analysis.

## Figures and Tables

**Table 1:** Demographic information of liner hosts (revision and postmortem).

Implant source (*N*)	Gender (*N*)	Side (*N*)	In situ time (mo.)	Cause of failure (*N*)
Revisions (54)	Females (22) Males (26) Unknown (6)	Left (24) Right (23) Unknown (7)	Range (1–108) Mean (26) Unknown (16)	Infection (10) Maltracking (9) Loose (9) Instability (5) Synovitis (2) Fracture (1) Osteolysis (1) Failed liner (1) PE wear^*∗∗*^ (1) Unknown (15)

Postmortem (21)	Females (13) Males (8)	Left (11) Right (10)	Range (19–144) Mean (79)	Autopsy (21)

Simulator (6)	Not applicable	Left (6)	60 months^*∗*^	Not applicable

Heavily delaminated (10)	Females (5) Males (4) Unknown (1)	Left (7) Right (3)	Range (24–130) Mean (73) Unknown (3)	Instability (2) Polyethylene wear (2) Tibial subsidence (2) Painful tibial component (1) Unknown (3)

^*∗*^1 million cycles representing 12 months of level walking.

^*∗∗*^PE = polyethylene.

**Table 2:** Summary of geometric parameters for retrieved and simulator components. Bold values denote a significant (*p* < 0.05) and meaningful (*R* ^2^ > 0.4) association with input variables “time in host” and “age at surgery.”

Mean (StDev)	Medial				Lateral			
Cluster number	Area (mm^2^)	Perimeter (mm)	ML stretch (mm)	AP stretch (mm)	Area (mm^2^)	Perimeter (mm)	ML stretch (mm)	AP stretch (mm)
1	**391.84** (135.11)	**79.18** (13.54)	23.59 (3.51)	**21.87** (6.03)	460.96 (166.04)	84.25 (12.78)	−12.77 (20.80)	25.64 (4.97)
2	498.21 (78.26)	83.56 (4.40)	24.39 (4.66)	26.15 (3.32)	566.35 (80.80)	89.47 (2.84)	12.25 (28.34)	27.06 (1.99)
3	712.27 (185.35)	100.02 (12.20)	26.87 (2.88)	32.92 (5.23)	754.24 (180.98)	**102.73** (11.72)	0.93 (29.67)	**33.26** (5.72)
4	416.07 (146.55)	78.78 (1.51)	23.12 (3.37)	23.68 (4.87)	421.97 (165.74)	79.01 (12.56)	−3.67 (26.80)	22.86 (6.32)
5^**∗**^	283.47	62.59	22.63	16.71	337.01	67.39	−20.51	21.38
6	418.87 (101.98)	77.46 (6.04)	21.78 (0.63)	24.22 (3.62)	412.37 (121.97)	76.86 (10.95)	−23.54 (2.06)	22.74 (3.61)
7^**∗**^	355.69	71.44	19.39	23.58	436.42	76.99	−26.19	21.71
8	179.84 (34.29)	54.08 (6.05)	17.21 (1.75)	14.83 (3.31)	143.83 (76.49)	46.72 (12.10)	3.17 (15.72)	13.87 (3.19)
9	374.52 (108.82)	75.28 (9.46)	23.33 (3.76)	21.05 (4.33)	392.90 (124.16)	74.99 (10.18)	3.62 (23.54)	21.97 (5.55)
10	363.73 (15.08)	73.69 (0.83)	22.83 (3.82)	21.21 (5.66)	308.54 (46.11)	68.10 (6.71)	−7.98 (28.02)	16.16 (3.08)
11	129.36 (88.82)	45.12 (18.78)	13.76 (5.50)	13.53 (6.04)	**241.70** (136.61)	58.06 (26.35)	8.61 (15.45)	16.62 (7.67)

StDev = standard deviation, ML stretch = medial-lateral stretch, and AP stretch = anterior-posterior stretch.

^**∗**^StDev not available, *n* (cluster) = 1.
